# Scale-free bursting activity in shrinkage induced cracking

**DOI:** 10.1038/s41598-024-57368-5

**Published:** 2024-03-26

**Authors:** Roland Szatmári, Akio Nakahara, So Kitsunezaki, Ferenc Kun

**Affiliations:** 1 Department of Theoretical Physics, Doctoral School of Physics, Faculty of Science and Technology, University of Debrecen, P.O.Box: 400, Debrecen, 4002 Hungary; 2 Laboratory of Physics, College of Science and Technology, Nihon University, 7-24-1 Narashinodai, Funabashi, 274-8501 Japan; 3 Research Group of Physics, Division of Natural Sciences, Nara Women’s University, Nara, 630-8506 Japan; 4 Institute for Nuclear Research (Atomki), P.O. Box 51, Debrecen, 4001 Hungary

**Keywords:** Nonlinear phenomena, Surfaces, interfaces and thin films, Nonlinear phenomena, Surfaces, interfaces and thin films

## Abstract

Based on computer simulations of a realistic discrete element model we demonstrate that shrinkage induced cracking of thin layers of heterogeneous materials, generating spectacular crack patterns, proceeds in bursts. These crackling pulses are characterized by scale free distributions of size and duration, however, with non-universal exponents depending on the system size and shrinking rate. On the contrary, local avalanches composed of micro-cracking events with temporal and spatial correlation are found to obey a universal power law statistics. Most notably, we demonstrate that the observed non-universality of the integrated signal is the consequence of the temporal superposition of the underlying local avalanches, which pop up in an uncorrelated way in homogeneous systems. Our results provide an explanation of recent acoustic emission measurements on drying induced shrinkage cracking and may have relevance for the acoustic monitoring of the electro-mechanical degradation of battery electrodes.

## Introduction

Under steady external driving a broad class of disordered systems exhibits an intermittent response evolving through a bursting sequence of the nucleation and growth of cracks^1^, collapse of voids^2,3^, motion of magnetic domain walls^4^ or dislocations^5^, rearrangement of particles^6^, firing of neurons^7^, etc. Experimental and theoretical investigations have revealed a scale-free statistics of crackling noise with a high degree of robustness across a broad range of length scales^8–10^. As a specific type of slowly driven systems, shrinkage induced cracking is ubiquitous in nature typically occurring when a material layer gradually dries or cools while attached to a substrate. The slowly evolving stress field leads to spectacular crack patters which can be observed from drying lake beds^11^, through the polygonal ground patterns in permafrost regions of Earth and Mars^12^, to the formation of columnar joints in cooling lava^11,13–15^. For industrial applications shrinkage induced cracking, on the hand, has a high potential making possible the structural control of crack patterns^16,17^, on the other hand, however, it may be undesired since it can lead to quality reduction of products^18^. In particular, restrained shrinking of solidifying dense pastes such as concrete is accompanied by the accumulation of cracks which may lead to strength reduction, with additional long lasting consequences due to the penetration of corrosive agents into construction components^19,20^. Recently, the application of the acoustic emission technique revealed that the crackling noise generated in drying concrete has scale-free statistics with a changing power law exponent as shrinking proceeds^19,20^. A similar cracking mechanism occurring in the electrode material has been found to be responsible for the capacity fading of e.g. Li-ion batteries during repeated charging-discharging cycles^21^. The acoustic monitoring of the bursts of electrode cracking proved to be a promising non-destructive technique to follow the electro-mechanical degradation process^22,23^. Experimental and theoretical studies of the past decades focused mainly on the structure of the emerging crack pattern^11,13,24,25^, however, understanding the emergence of shrinkage induced crackling noise, and its statistical and dynamical properties still remained an open problem of fundamental importance.

Here we study this problem by means of a realistic discrete element model (DEM) of a thin material layer which undergoes restrained shrinking while attached to a rigid substrate. Based on computer simulations we show that cracking of the layer proceeds in bursts whose magnitude and duration obey scale-free statistics in agreement with the experimental findings. However, we demonstrate that power law exponents are not universal, in particular, they depend on the sample size and on the driving rate. Our calculations revealed that the non-universal behaviour of crackling noise exponents can be understood in terms of the temporal superposition of local avalanches of growing cracks, which are characterized by universal distributions. Our results provide a possible explanation of the experimental findings with important implications for testing and assessing of construction materials whose manufacturing involves solidification accompanied by shrinking, and for the acoustic monitoring of battery degradation.

## Results

We use a discrete element model (DEM) to study the cracking process of a thin material layer while it shrinks attached to a rigid substrate. The analysis of the temporal evolution of the system revealed an intermittent cracking activity which corresponds to the acoustic outbreaks of real experiments.

### Discrete element simulations of the breakup of a shrinking layer

**Figure 1:**
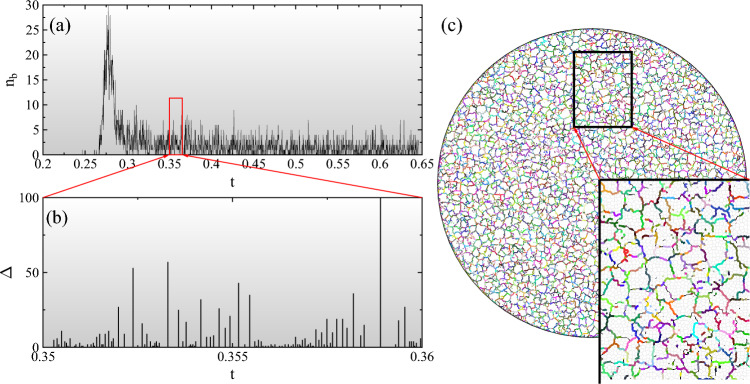
Temporal evolution (**a**, **b**) and spatial distribution (**c**) of shrinking induced cracking for a system of radius $R=120$ (in unit of the average polygon size $l_0$). In (**a**) the number of breaking beams $n_b$ is plotted as a function of time *t*. (**b**) demonstrates for a small segment of the time series that the breaking activity of the system can be decomposed into distinct pulses the size of which $\Delta$ is plotted at the time of their occurrence. A snapshot of the cracking layer is presented in (**c**) after the connected crack network emerged. Micro-cracks are colored according to the local avalanche of breakings they belong to. A magnified view in the bottom-right corner reveals that local avalanches are intermittent steps of the growth of cracks.

To mimic the typical experimental conditions, in the model a disc-shaped specimen of radius *R* is considered, which is discretized on a random lattice of space filling convex polygons representing mesoscopic pieces of the layer (see Fig. 1(c) for illustration). Polygons adjacent to each other in the initial tessellation are connected by breakable elastic beams of Young modulus *E*. Shrinking is represented by gradually reducing the natural length of beams with a fixed rate *s*, which results in a linearly increasing homogeneous strain $\varepsilon =st$ as time *t* elapses. To capture the adhesion of the layer to a substrate, the middle point of the polygons is attached to the underlying plane by an elastic spring of stiffness $D_s$, which is stress free in the initial configuration. As a consequence of the restrained shrinking of the layer, beams get overstretched and break according to a physical breaking rule (see Methods for details). Adhesion springs do not break so that no delamination of the layer can occur in the model.

To generate the time evolution of the particle system we carry out discrete element simulations solving the equation of motion of the polygons^26,27^ (see Methods). The breaking criterion is evaluated in each iteration step and those beams which fulfil the condition are removed from the system. Cracks are formed by adjacent broken beams along the edges of polygons. The model naturally captures the simultaneous propagation and interaction of cracks, which eventually leads to the breakup of the layer into fragments. A snapshot of the evolving crack pattern is presented in Fig. 1c, where for clarity the beams are not shown. Further details of the model construction and the value of material parameters can be found in Ref.^28^. The model has been applied to study the shrinkage induced cracking of a layer, where it successfully reproduced the experimental findings on the structure of the planar crack pattern, and on the shape and statistics of the mass of fragments^28,29^.

### Crackling noise during shrinkage induced cracking

**Figure 2:**
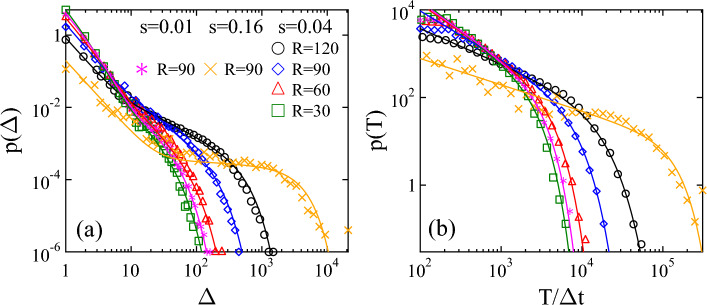
Probability distributions of the size $p(\Delta )$ (**a**) and duration *p*(*T*) (**b**) of cracking pulses for several different system sizes *R* and strain rates *s*. Continuous lines represent fits with Eq. (1) (**a**) and with Eq. (2) (**b**). In (**b**) $\Delta t$ denotes the time step of DEM simulations.

As the layer shrinks cohesive contacts break and result in the nucleation of micro-cracks along the common edge of adjacent polygons. The breaking thresholds have fixed values for all the beams, however, the random tessellation introduces structural disorder into the material, which gives rise to a random nucleation of micro-cracks in the homogeneous deformation field of the layer. To characterize the temporal evolution of the cracking process, for each broken beam we record its position $\textbf{r}_i^b$ and breaking time $t_i^b$ from which the entire damage evolution of the layer can be reconstructed. In experiments crackling noise sensors like acoustic emission (AE) devices accumulate the effect of elastic waves generated by simultaneous cracking over extended spatial regions of the specimen^30^. Hence, we characterize the overall evolution of the breaking activity of the shrinking layer by the number of micro-cracks $n_b$ that occurred in equally sized time bins, which is plotted in Fig. 1a as a function of time *t*. The sudden rise and peak of the $n_b(t)$ curve mark the point of the dynamics where the deformation becomes sufficiently high to drive the growth of nucleated cracks in random directions. Growing cracks gradually merge and form a connected crack network along which the layers falls apart into a large number of fragments. Further shrinking results in cracks inside fragments splitting them into two pieces. For snapshots of the time evolution of the cracking layer see Fig. S1 of Supplementary Note 1. Figure 1c demonstrates that the emerging crack network has an isotropic cellular structure resulting in a nearly polygonal shape of the fragments^28^ in agreement with measurements on various types of shrinking induced cracking phenomena^31,32^. The stochastic nature of the $n_b(t)$ curve in Fig. 1a indicates that in spite of the slow homogeneous increase of the deformation of the layer, damage accumulation through the nucleation and growth of cracks proceeds in an intermittent way. In order to identify bursts of the breaking activity we introduce a correlation time $t_c$ and assume that two consecutive micro-cracks (beam breakings) of time $t_i^b$ and $t_{i+1}^b$ belong to the same trail of breakings if they follow each other within $t_c$, i.e. if the condition $t_{i+1}^b-t_i^b<t_c$ holds. This way the temporal sequence of micro-cracks is decomposed into bursts, i.e. pulses of cracking activity with a large number of breakings separated by silent periods where no breaking occurs. This is demonstrated in Fig. 1b where pulses of a small segment of the time series of Fig. 1a are highlighted. The size $\Delta$ of a pulse is the number of beams failed in the trail of breaking events, the pulse duration *T* follows as the difference between the time of the last $t^b_{last}$ and first $t^b_{first}$ beam breaking $T=t^b_{last}-t^b_{first}$, while the waiting time $t_W$ between consecutive pulses is the duration of the silent period between them. These pulses of the global cracking activity are analogous to the acoustic outbreaks of real experiments on restrained shrinking so that the statistics of pulse quantities can be compared to the corresponding results of AE measurements^19,20,22,23^. The probability distribution $p(\Delta )$ of pulse sizes is presented in Fig. 2a for four different system sizes *R* at a fixed shrinking rate *s* along with two additional data sets at a lower and a higher value of *s* for $R=90$.

A highly complex behaviour of $p(\Delta )$ is observed: at the smallest system size $R=30$ the size distribution $p(\Delta )$ exhibits a power law decrease followed by an exponential cutoff. However, for larger systems pulses grow to larger sizes and a crossover occurs to a second power law regime of a lower exponent for large pulses, while the slope of the curve remains practically constant in the regime of small $\Delta$. Increasing the shrinking rate *s* at a fixed system size *R* has a similar effect, i.e. for higher *s* larger pulses are obtained accompanied by a crossover to a second power law of a lower exponent. To give a quantitative description of the evolution of the pulse size distribution $p(\Delta )$ we use the functional form

1 $$\begin{aligned} p(\Delta ) = A \Delta ^{-\tau _l}e^{-\Delta /\Delta _l} +B\Delta ^{-\tau _h}e^{-\left( \Delta /\Delta _h\right) ^{\delta }}, \end{aligned}$$

to fit the curves in Fig. 2a. In Eq. (1) we assume that the size distribution $p(\Delta )$ is the sum of two power laws with exponential cutoffs, where $\tau _l$ and $\tau _h$ are the exponents in the low and high pulse size regimes, the corresponding cutoff sizes are denoted by $\Delta _l$ and $\Delta _h$, while the parameters *A* and *B* control the relative contributions of the two terms in the distribution. The continuous lines in Fig. 2a represent fits with Eq. (1) obtained in such a way that $\tau _l$ was fixed to the value $\tau _l=1.95\pm 0.05$ of $R=30$, where the distribution is uni-modal without crossover, using only the other exponent $\tau _h$ as a fitting parameter. It can be observed that the value of $\tau _h$ decreases from the vicinity of $\tau _h=1.95\pm 0.06$ towards 0 as *R* and *s* increase. The exponent $\delta$ controls the shape of the cutoff of $p(\Delta )$ having the same value $\delta =1.50\pm 0.06$ in all cases. Figure 2b demonstrates that the probability distribution of the duration *p*(*T*) undergoes a similar evolution, i.e. in the regime of large durations, *p*(*T*) can always be approximated by a single power law followed by an exponential cutoff

2 $$\begin{aligned} p(T) \sim T^{-\alpha }e^{-\left( T/T_0\right) ^{\delta }}, \end{aligned}$$

however, increasing *R* and *s* the cutoff duration $T_0$ shifts to higher values, while the power law exponent $\alpha$ gradually decreases from $\alpha =1.4\pm 0.06$ to $\alpha =0.8\pm 0.04$ in the parameter regime considered. The value of the cutoff exponent $\delta$ varies between 1.5 and 2.0. The waiting time distribution $p(t_W)$ characterizes the statistics of silent periods between consecutive pulses. Figure 3 in Supplementary Note 3 presents that $p(t_W)$ has a functional form similar to the duration distributions, i.e. a power law functional form is obtained with an exponential cutoff. Increasing the shrinking rate *s* and the system size *R* the time gap between consecutive pulses decreases, hence, the exponent *z* of the power law regime of $p(t_W)$ increasis while the characteristic waiting time controlling the cutoff decreases (see Supplementary Note 3).

### Temporal superposition of local avalanches

In order to understand the origin of the complex evolution of pulse statistics with the system size *R* and driving rate *s*, it is important to emphasize that arranging micro-cracking events into pulses of activity is strictly based on the temporal sequence of micro-cracks. This is to mimic that in real experiments acoustic sensors integrate the signal of nucleating and propagating cracks over extended spatial regions which may involve even the entire specimen^30,33,34^. Hence, it can be expected that pulses observed in the shrinking layer emerge as the superposition of a large number of uncorrelated local avalanches of micro-cracks which are randomly scattered all over the sample. To prove that this mechanism is indeed responsible for the observed non-universality, we identify local avalanches as temporally and spatially correlated trails of micro-cracks, which have to be strictly distinguished from global activity pulses. Local avalanches always start from a single breaking bond and evolve as a spatially connected set of micro-cracks which follow each other within the correlation time $t_c$ and length $l_c$ according to the conditions $t_{i+1}^b-t_i^b < t_c$ and $\left| \textbf{r}_{i+1}^b-\textbf{r}_i^b\right| <l_c$. In the calculations $l_c$ was set to be the double of the average polygon size $l_0$ of the discretization, while $t_c$ is the characteristic time scale of load redistribution determined by the material properties of the layer. Computer simulations revealed that local avalanches identified by the algorithm are intermittent steps of growing cracks spanning between junction points of the crack network (see Fig.  1c).

**Figure 3:**
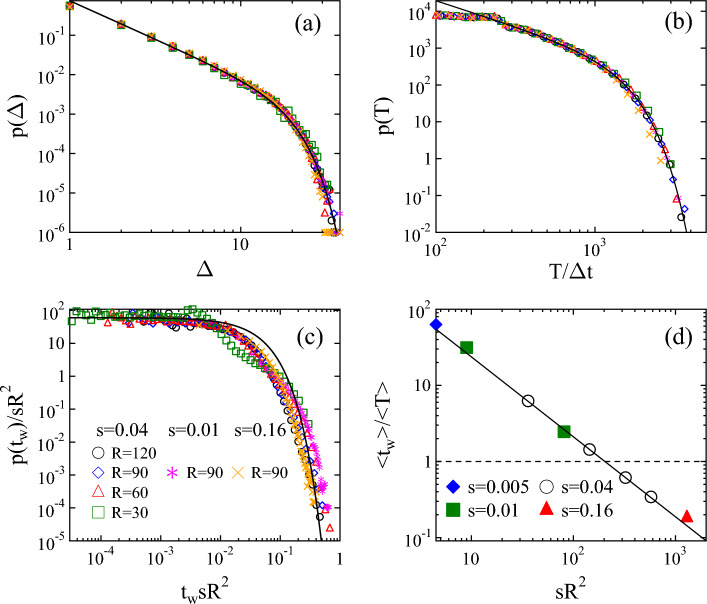
Probability distribution of the size $p(\Delta )$ (**a**), duration *p*(*T*) (**b**), and waiting time $p(t_W)$ (**c**) of local avalanches for several system sizes *R* and strain rates *s*. (**c**) demonstrates that the waiting time distributions $p(t_W)$ rescaled with the product $sR^2$ fall on the top of each other. The bold line represents an approximate exponential function. (**d**) Ratio of the average waiting time $\left<t_W\right>$ and the average duration $\left<T\right>$ of local avalanches as a function of $sR^2$. Results obtained at different *R* and *s* values fall on the same decreasing power law of exponent 1 indicated by the continuous line.

It can be observed in Fig. 3(a,b) that both the size $\Delta$ and duration *T* of local avalanches are power law distributed with an exponential cutoff. It is important to emphasize that local avalanches exhibit a high degree of robustness, i.e. for both distributions $p(\Delta )$ and *p*(*T*) the results obtained at different system sizes *R* and driving rates *s* fall on a master curve. Although, the ranges spanned by $\Delta$ and *T* are limited, i.e. the cutoff avalanche size $\Delta _0$ and duration $T_0$ are smaller than their counterparts of pulses, the curves can be very well fitted by the functional form of Eq. (2) with the power law exponents $\tau _l=1.95\pm 0.06$ and $\alpha _l=1.4\pm 0.04$. Local avalanches are determined by the material properties of the shrinking layer such as the ratio of the stiffness of adhesion springs and beam elements $D_s/E$, which controls the characteristic length scale of stress heterogeneities in the system. Figure S2 of Supplementary Note 2 demonstrates that at lower values of $D_s/E$ local avalanches grow to larger sizes and durations, however, the exponents $\tau _l$ and $\alpha _l$ remain the same.

Due to the quenched structural disorder of the material, the homogeneous deformation field gives rise to a random pop up of local avalanches without any temporal correlation as the layer shrinks. It follows that the temporal occurrence of local avalanches can be approximated by a Poissonian process, where the effect of the system size *R* and of the driving rate *s* is that in a larger system a higher number of weak spots are available where cracks can nucleate, and at higher strain rates *s* the deformation increments $s\cdot \Delta t$ needed to trigger the next local avalanche is faster reached, respectively. As a consequence, in a homogeneous system the average nucleation rate $\left<r\right>$ of avalanches must be proportional to the volume, here the area, of the system $R^2$, and to the shrinking rate *s* so that $\left<r\right>\sim sR^2$ holds. Figure 3c demonstrates that rescaling the waiting time distributions $p(t_W)$ of local avalanches with $sR^2$, distributions obtained at different system sizes *R* and shrinking rates *s* collapse on a master curve. The scaling function can be well approximated by an exponential as it is expected for Poissonian processes.

**Figure 4:**
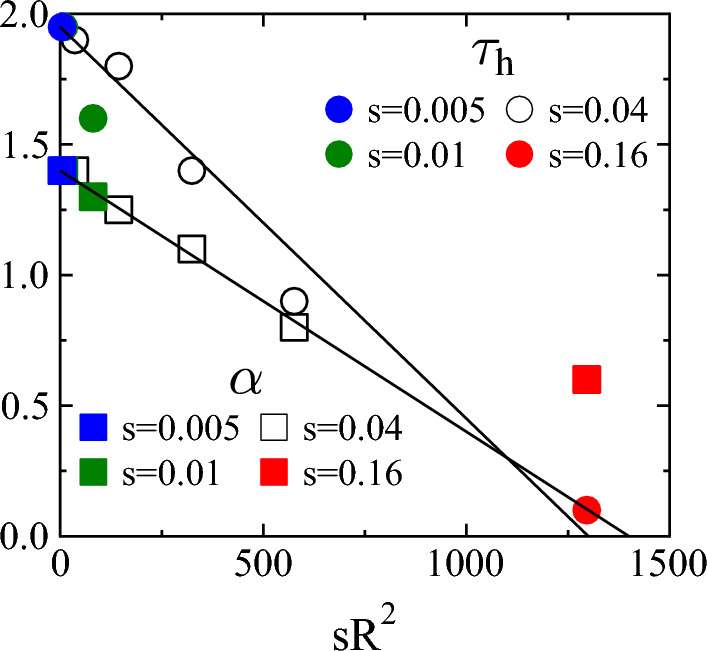
Power law exponents $\tau _h$ and $\alpha$ of the probability distribution of the size $p(\Delta )$ and duration *p*(*T*) of cracking pulses as function of $sR^2$. For both exponents an approximate linear dependence is obtained on the nucleation rate of local avalanches described by Eqs. (3, 4). Note that the number of pulses decreases with increasing nucleation rate $sR^2$. As a consequence the error bars of the exponents increase from 0.04 to 0.15 from the lowest to the highest values of $sR^2$.

The results imply that the non-universal statistics of global activity pulses is caused by the superposition of temporally overlapping local avalanches of cracking, which randomly pop up in the system without any correlation. The relevance of overlapping can be quantified by the ratio of the average waiting time $\left<t_W\right>$ and of the average duration $\left<T\right>$ of local avalanches. Figure 3d demonstrates that plotting the ratio $\left<t_W\right>/\left<T\right>$ as a function of the product $sR^2$, results of all the simulations fall on a decreasing power law of exponent 1 implying $\left<t_W\right> \sim 1/sR^2 \sim 1/\left<r\right>$, which confirms the homogeneous Poissonian statistics of the temporal occurrence of local avalanches. It can also be inferred from the figure that at sufficiently small system sizes *R* and strain rates *s* the average waiting time can be significantly larger than the avalanche duration $\left<t_W\right>/\left<T\right>\gg 1$, which implies that local avalanches form a sequence of well separated events without hardly any possibility of overlap in time. However, the value $\left<t_W\right>/\left<T\right>\ll 1$ obtained for large *R* and *s* indicates a strong temporal overlap of local events. For very large values of the product $sR^2$ this overlap can be so dominating that practically all local avalanches may get superimposed into a single or just a few pulses. This is supported by the evolution of the waiting time distribution $p(t_W)$ of pulses with the system size *R* and driving rate *s*, see Fig. S3 of Supplementary Note 3. Based on our numerical results, we assume a linear dependence of the characteristic exponents $\tau _h$ and $\alpha$ of the distributions of pulse quantities on the nucleation rate $\left<r\right>$ of local avalanches

3 $$\begin{aligned} \tau _h&= \tau _l-a\left<r\right>, \end{aligned}$$

4 $$\begin{aligned} \alpha&= \alpha _l - b\left<r\right>, \end{aligned}$$

where *a* and *b* are constants that can be obtained by fitting. Note that Eqs. (3, 4) capture the fact that in the limit of zero driving $\left<r\right>\rightarrow 0$ the exponents $\tau _h$ and $\alpha$ must converge to the corresponding exponents $\tau _l$ and $\alpha _l$ of local avalanches. Figure 4 demonstrates that the linear form Eqs. (3, 4) provides a reasonable description of the joint effect of the system size *R* and shrinking rate *s* on the exponents of the integrated signal with $a=0.0015\pm 0.0003$ and $b=0.0010\pm 0.0004$ so that the ratio of the two parameters *a* and *b* is $a/b\approx 1.5$.

## Discussion

We showed that the restrained shrinking of a homogeneous layer of disordered materials gives rise to an intermittent evolution of cracking which can be decomposed into pulses of activity separated by silent periods. The size and duration of pulses have a scale-free statistics, similarly to other crackling phenomena, however, with non-universal exponents depending on the system size and on the driving rate. Our calculations revealed an astonishing universal micro-scale process underlying the non-universal behaviour of the integrated signal. Most notably, we demonstrated that the local avalanches of micro-cracking events are characterized by universal scaling laws. The homogeneity of the deformation field and the uncorrelated spatial disorder of the layer give rise to a Poissonian process of the temporal occurrence of local avalanches. The observed non-universality of the global cracking activity is caused by the superposition of temporally overlapping local avalanches of micro-cracks. The power law exponents of the distributions of pulse sizes and durations proved to decrease linearly from the corresponding exponents of local avalanches towards zero as the nucleation rate of avalanches increases.

The number of systematic acoustic emission studies on shrinking induced cracking is rather limited in the literature. AE measurements on shrinking concrete revealed non-universal power law exponents increasing as the sample dries^19,20^. Our calculations suggest that the decreasing shrinking rate during solidification decreases the nucleation rate of local cracking events, which reduces their temporal overlap, and hence, increases the exponents towards their counterparts of local avalanches. Intermittent growth of shrinking induced cracks has also been observed during the formation of columnar joints, e.g. for the Kilauean lava lakes by recording seismic waves^35^. However, no systematic data is available on the statistics of cracking events. In general, the chisel-like marks often observed on the surface of basalt columns, called striae, are traces of rapid advancement of the crack front triggered by slow shrinking^36,37^. Based on the simulation results we conjecture that the area of striae is composed of local avalanches of the crack front so that its distribution may depend on the cooling rate of lava which should be tested by field measurements. A somewhat similar situation has been observed in crack propagation measurements where global avalanches of the advancing front of a planar crack have been found to be composed of clusters of locally failing elements along the front, which can be identified e.g. by complementing AE measurements using high speed imaging techniques^38,39^. A non-universal behaviour of crackling activity was also observed in the case of Barkhausen noise where the superposition of local avalanches of the motion of magnetic domain walls was found to be responsible for the driving rate dependence of crackling noise exponents. Varying the rate of change of the driving magnetic field a linear behaviour of the size and duration exponents was found^40^.

During charging-discharging cycles of e.g. Li-ion batteries the lithiation-delithiation process causes volume change of the electrode material which eventually gives rise to cracking accompanied by acoustic outbreaks^22,23^. Our results can help to decipher the information encoded in the acoustic event series measured at different rates of charging-discharging, which may serve as a basis for the assessment of the state of batteries in operation.

## Methods

*Discrete element model:* In the model a disc-shaped specimen of radius *R* is discretized in terms of randomly shaped convex polygons obtained by a Voronoi construction. We use a regularised Voronoi tessellation, i.e. first a square lattice is placed on the sample and basis points of the tessellation are thrown randomly and independently into the plaquettes of the lattice. This technique results in a well-defined average polygon size $l_0$, which is used as the unit length of the model. Polygons adjacent to each other in the initial tessellation are connected by breakable elastic beams of Young modulus *E*. The length and cross-section of beams are determined by the geometry of the polygonal lattice, which introduces randomness into their physical properties, e.g. moduli and moments^28^.

Shrinking of the layer is captured by gradually reducing the natural length of beams with a fixed rate *s*, which results in a linearly increasing homogeneous strain $\varepsilon =st$ as time *t* elapses. To take into account the adhesion of the layer to a substrate, the middle point of the polygons is attached to the underlying plane by an elastic spring of stiffness $D_s$, which is stress free in the initial configuration. During the restrained shrinking of the layer the beam elements get overstretched and break according to the breaking rule

5 $$\begin{aligned} \left( \frac{\varepsilon _{ij}}{\varepsilon _{th}}\right) ^2 + \frac{\text{ max }(|\Theta _i|, |\Theta _j|)}{\Theta _{th}} \ge 1, \end{aligned}$$

where $\varepsilon _{ij}$ denotes the longitudinal strain of the beam connecting polygons *i* and *j*, while $\Theta _i$ and $\Theta _j$ are the bending angles of the two beam ends^28^. Eq. (5) takes into account that stretching and bending contribute to the breaking of beams and the relative importance of the breaking modes is controlled by the breaking thresholds $\varepsilon _{th}$ and $\Theta _{th}$. The breaking parameters $\varepsilon _{th}$ and $\Theta _{th}$ have fixed values for all the beams so that the structural randomness introduced by the random discretization is the only source of disorder in the system. Adhesion springs are not allowed to break so that no delamination can occur in the model. Those polygons which are not connected by beams may overlap during their motion, and we apply a repulsive force between them proportional to the overlap area.

To generate the time evolution of the particle system we carry out discrete element simulations solving the equation of motion of the polygons for the translational and rotational degrees of freedom^26,27^. For the numerical solution we use a 5*th* order Predictor-Corrector scheme which provides sufficient precision and stability. To mimic the effect of sticking to the container wall, polygons along the circular boundary of the layer are fixed during the simulations. Further details of the model construction and the value of its parameters can be found in Ref.^28^. The model has been applied to study the shrinkage induced cracking of a layer, where it successfully reproduced the experimental findings on the structure of the planar crack pattern, and on the shape and statistics of the mass of fragments^28,29^. In the present simulations the sample radius *R* was varied in the range $30\le R \le 120$ so that the number of polygons of discs falls between $\sim 3.000$ and $\sim 45.000$.

### Supplementary Information

Supplementary Information.

## Data Availability

The datasets generated and analysed during the current study are available from the corresponding author on reasonable request.

## Code availability

The numerical code used for data evaluation in this paper is available from the corresponding author upon reasonable request.

## References

[1] Laurson L (2013). Evolution of the average avalanche shape with the universality class. Nat. Commun..

[2] Nataf GF (2014). Avalanches in compressed porous SiO2-based materials. Phys. Rev. E.

[3] Salje EKH (2018). Intermittent flow under constant forcing: Acoustic emission from creep avalanches. Appl. Phys. Lett..

[4] Zapperi S, Castellano C, Colaiori F, Durin G (2005). Signature of effective mass in crackling-noise asymmetry. Nat. Phys..

[5] Ispánovity PD (2014). Avalanches in 2d dislocation systems: Plastic yielding is not depinning. Phys. Rev. Lett..

[6] Liu C, Ferrero EE, Puosi F, Barrat J-L, Martens K (2016). Driving rate dependence of avalanche statistics and shapes at the yielding transition. Phys. Rev. Lett..

[7] Shriki O (2013). Neuronal avalanches in the resting meg of the human brain. J. Neurosci..

[8] Davidsen J, Stanchits S, Dresen G (2007). Scaling and universality in rock fracture. Phys. Rev. Lett..

[9] Baró J (2013). Statistical similarity between the compression of a porous material and earthquakes. Phys. Rev. Lett..

[10] Sethna JP, Dahmen KA, Meyers CR (2001). Crackling noise. Nature.

[11] Goehring, L., Nakahara, A., Dutta, T., Kitsunezaki, S. & Tarafdar, S. *Desiccation Cracks and their Patterns: Formation and Modelling in Science and Nature* (John Wiley & Sons, 2015).

[12] Forte E, French HM, Raffi R, Santin I, Guglielmin M (2022). Investigations of polygonal patterned ground in continuous antarctic permafrost by means of ground penetrating radar and electrical resistivity tomography: Some unexpected correlations. Permafr. Periglac. Process..

[13] Aydin A, Degraff JM (1988). Evoluton of polygonal fracture patterns in lava flows. Science.

[14] Lamur A (2018). Disclosing the temperature of columnar jointing in lavas. Nat. Commun..

[15] Bourdin B, Marigo J-J, Maurini C, Sicsic P (2014). Morphogenesis and propagation of complex cracks induced by thermal shocks. Phys. Rev. Lett..

[16] Nam KH, Park IH, Ko SH (2012). Patterning by controlled cracking. Nature.

[17] Guo L, Ren Y, Kong LY, Chim WK, Chiam SY (2016). Ordered fragmentation of oxide thin films at submicron scale. Nat. Commun..

[18] Qureshi TS, Al-Tabbaa A (2016). Self-healing of drying shrinkage cracks in cement-based materials incorporating reactive MGO. Smart Mater. Struct..

[19] Xia Q (2017). Cracking behaviour of restrained cementitious materials with expansive agent by comprehensive analysis of residual stress and acoustic emission signals. Adv. Cem. Res..

[20] Bacharz M, Bacharz K, Trampczynski W (2022). The correlation between shrinkage and acoustic emission signals in early age concrete. Materials.

[21] Christensen J, Newman J (2006). Stress generation and fracture in lithium insertion materials. J. Solid State Electrochem..

[22] Schweidler S, Dreyer SL, Breitung B, Brezesinski T (2021). Operando acoustic emission monitoring of degradation processes in lithium-ion batteries with a high-entropy oxide anode. Sci. Rep..

[23] Lee S-M, Kim J-Y, Lee J, Byeon J-W (2023). Evaluation of cracking damage in electrode materials of a lmo/al-lix lithium-ion battery through analysis of acoustic emission signals. J. Market. Res..

[24] Hofmann M, Anderssohn R, Bahr H-A, Weiß H-J, Nellesen J (2015). Why hexagonal basalt columns?. Phys. Rev. Lett..

[25] Domokos G, Jerolmack DJ, Kun F, Török J (2020). Plato’s cube and the natural geometry of fragmentation. Proc. Natl. Acad. Sci..

[26] Allen MP, Tildesley DJ (2002). Computer simulation of Liquids.

[27] Alava M, Nukala PK, Zapperi S (2006). Statistical models of fracture. Adv. Phys..

[28] Szatmári R, Halász Z, Nakahara A, Kitsunezaki S, Kun F (2021). Evolution of anisotropic crack patterns in shrinking material layers. Soft Matter..

[29] Halász Z, Nakahara A, Kitsunezaki S, Kun F (2017). Effect of disorder on shrinkage-induced fragmentation of a thin brittle layer. Phys. Rev. E.

[30] Stojanova M, Santucci S, Vanel L, Ramos O (2014). High frequency monitoring reveals aftershocks in subcritical crack growth. Phys. Rev. Lett..

[31] Nakahara A, Hiraoka T, Hayashi R, Matsuo Y, Kitsunezaki S (2019). Mechanism of memory effect of paste which dominates desiccation crack patterns. Phil. Trans. R. Soc. A.

[32] Kitsunezaki S, Nakahara A, Matsuo Y (2016). Shaking-induced stress anisotropy in the memory effect of paste. Europhys. Lett..

[33] Maloy KJ, Santucci S, Schmittbuhl J, Toussaint R (2006). Local waiting time fluctuations along a randomly pinned crack front. Phys. Rev. Lett..

[34] Mair K, Main I, Elphick S (2000). Sequential growth of deformation bands in the laboratory. J. Struct. Geol..

[35] Peck DL, Minakami T (1968). The Formation of Columnar Joints in the Upper Part of Kilauean Lava Lakes. Hawaii. GSA Bull..

[36] Goehring, L. & Morris, S.W. Scaling of columnar joints in basalt. *J. Geophys. Rese. Solid Earth* **113** (2008).

[37] Sakaguchi H, Yamada K, Shimokawa M (2022). Observation and a simple model of plumose pattern on fracture surfaces of drying pastes. EPL.

[38] Laurson L, Santucci S, Zapperi S (2010). Avalanches and clusters in planar crack front propagation. Phys. Rev. E.

[39] Santucci S (2019). Avalanches and extreme value statistics in interfacial crackling dynamics. Philos. Trans. R. Soc. Math. Phys. Eng. Sci..

[40] White RA, Dahmen KA (2003). Driving rate effects on crackling noise. Phys. Rev. Lett..

