# Supplementary material for: Scale-free bursting activity in shrinkage induced cracking
Source: Sci Rep. 2024 Mar 26;14:7101. doi: 10.1038/s41598-024-57368-5 (PMC10965922; doi:10.1038/s41598-024-57368-5)
Supplement: Supplementary file 1 — Supplementary Information. [file 41598_2024_57368_MOESM1_ESM.pdf]

Supplementary Information for

R. Szatmári[1], A. Nakahara[2], S. Kitsunezaki[3], and F. Kun[4,5]

# *Scale-free bursting activity in shrinkage induced cracking*

[1]Department of Experimental Physics, Doctoral School of Physics, Faculty of Science and Technology, University of Debrecen, P.O. Box 400, H-4002 Debrecen, Hungary

[2]Laboratory of Physics, College of Science and Technology, Nihon University, 7-24-1 Narashinodai, Funabashi, 274-8501, Japan

[3]Research Group of Physics, Division of Natural Sciences, Faculty of Nara Women's University, Nara, 630-8506, Japan

[4]Department of Theoretical Physics, Faculty of Science and Technology, University of Debrecen, P.O. Box 400, H-4002 Debrecen, Hungary

[5]Institute of Nuclear Research (Atomki), P.O. Box 51, H-4001 Debrecen, Hungary

## 1 Supplementary Note 1: Time evolution of the cracking layer

As the layer shrinks while attached to the substrate beams get overstretched and break when their local strength is exceeded. Due to the uncorrelated structural disorder of the layer, crack nucleation occurs at random locations all over the sample. The steady shrinking drives the growth of cracks, which merge and gradually form a connected crack network. This process is illustrated in Fig. 1, where the connected crack network first occurs in $(b)$. Along the cracks the layer falls apart into a large number of fragments (see Fig. $1(b)$). As shrinking proceeds cracks nucleate inside fragments which typically breaks them into two further pieces, giving rise to a binary fragmentation process. As a consequence the size of fragments gets gradually reduced, which can be observed in Figs. $1(b, c, d)$.

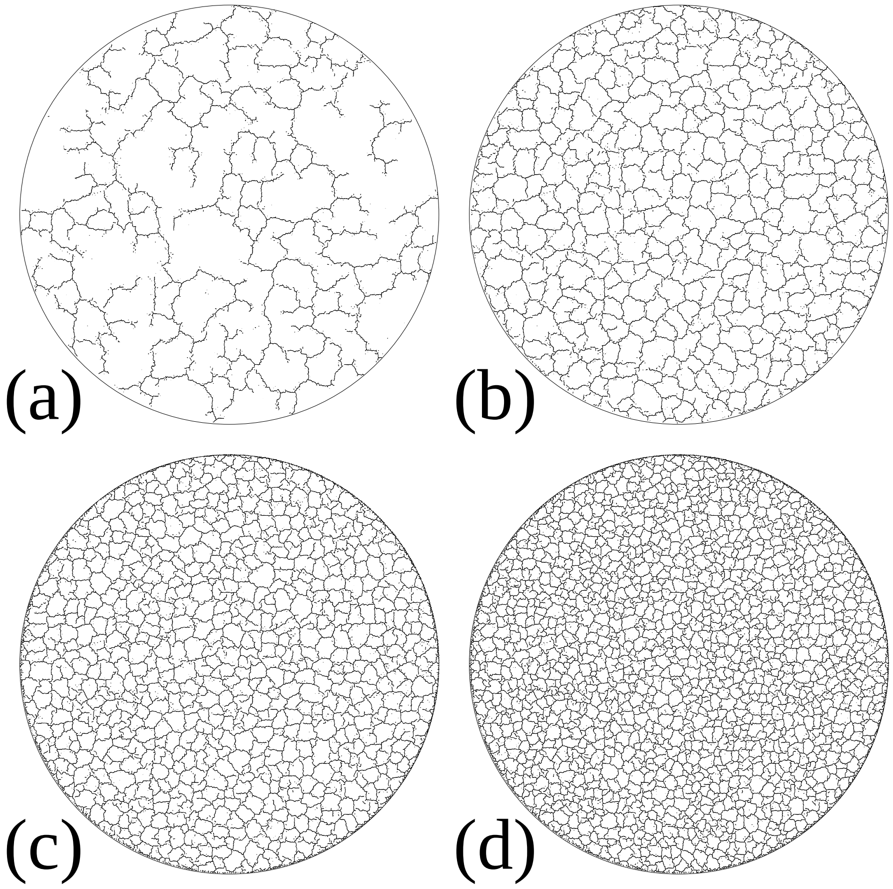


Supplementary Figure 1: Time evolution of the cracking layer. ($a$) After nucleation cracks grow and merge. The fully connected crack network is formed in ($b$) where a large number of fragments can be observed. ($c, d$) As shrinking proceeds, cracks nucleate inside fragments, which typically breaks the fragments into two pieces.

# 2 Supplementary Note 2: Dependence of local avalanches on material properties

Simulations revealed that local avalanches exhibit a high degree of robustness in the sense that the distributions of their size $p(\Delta)$ and duration $p(T)$ do not depend on the driving rate $s$ and on the system size $R$, they only depend on the material properties of the shrinking layer and on the strength of adhesion. Local avalanches are the abrupt jumps made by growing cracks, which

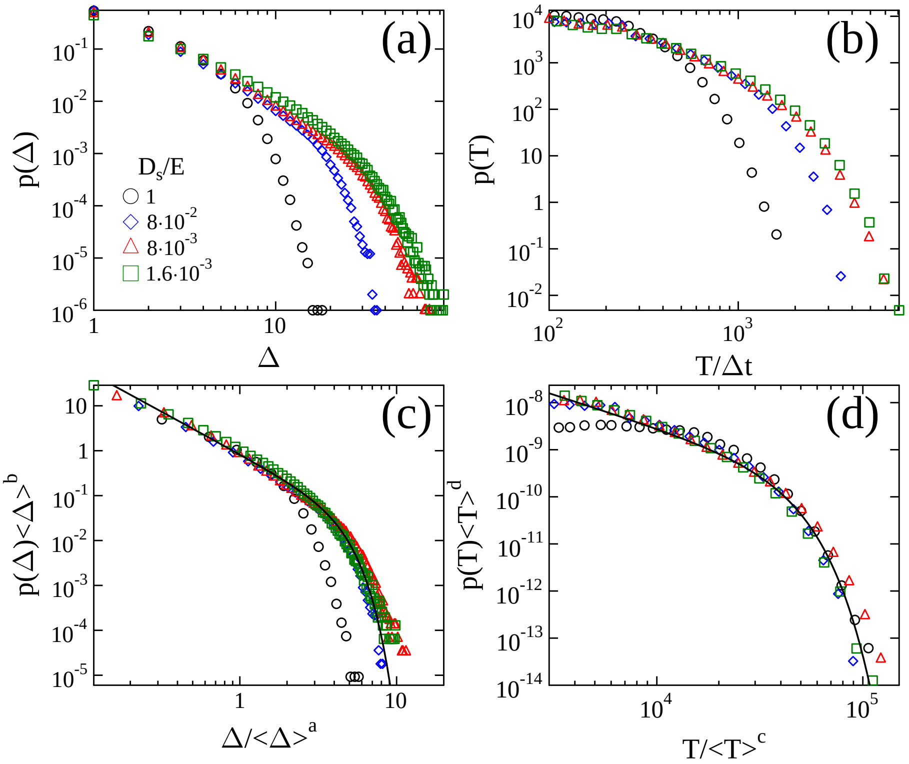


Supplementary Figure 2: Probability distribution of the size $p(\Delta)$ $(a)$ and duration $p(T)$ $(b)$ of local avalanches at different values of the ratio $D_s/E$. The corresponding scaling plots are presented in $(c)$ and $(d)$. Rescaling the distributions with proper powers of the average avalanche size $\langle\Delta\rangle$ $(c)$ and of the average duration $\langle T\rangle$ $(d)$, the curves obtained at different values of $D_s/E$ can be collapsed on the top of each other. In $(c)$ and $(d)$ the continuous lines represent best fits obtained with Eq. (S1).

are strongly affected by the characteristic length scale $l_r$ of stress inhomogeneities in the layer. In the model the value of $l_r$ is controlled by the ratio of the adhesion strength $D_s$ and of the Young modulus $E$ of beams. To explore this material dependence, we performed simulations at several values of $D_s$ at a fixed driving rate $s = 0.04$ and system size $R = 90$. The probability distributions of the size $p(\Delta)$ and duration $p(T)$ of the local avalanches are presented in Figs. $2(a, b)$. It can be observed that at lower values of the ratio $D_s/E$, where stress inhomogeneities extend over a larger distance, avalanches have a larger size $\Delta$ and a longer duration $T$ indicated by the larger cutoff values of the distributions. However, the functional form of the distributions

remains the same, i.e. in all cases power laws are obtained followed by an exponential cutoff. Note that the slope of the curves in the power law regime is constant, i.e. independent of the value of $D_s/E$. This universality of the exponents of the size $\tau_l$ and duration $\alpha_l$ of local avalanches is confirmed by the data collapse analysis presented in Figs. $2(c, d)$. The figures demonstrate that rescaling the distributions $p(\Delta)$ and $p(T)$ by proper powers of the corresponding average size $\langle\Delta\rangle$ and duration $\langle T\rangle$, the distributions obtained at different values of $D_s/E$ fall on a master curve. The quality of collapse is satisfactory, only the data set of the largest ratio $D_s/E$ shows some deviation from the others. The value of the scaling exponents $a, b, c, d$ providing best collapse are $a = 1.7$, $b = 3.3$, and $c = 2.0$, $d = 3.0$, for the avalanche size and duration, respectively. In both Figs. $2(c)$ and $(d)$ the scaling function obtained by the data collapse can be well fitted by the expression

$$p(x) \sim x^{-\eta} e^{-(x/x_0)^{\delta}}, \quad \text{(S1)}$$

where $x_0$ is the cutoff parameter and the exponent $\eta$ represents $\tau_l$ and $\alpha_l$. The value of the local avalanche exponents was found to be $\tau_l = 1.95 \pm 0.06$ and $\alpha_l = 1.40 \pm 0.04$. The exponent $\delta$ controlling the shape of the cutoff has the values $\delta = 2.7 \pm 0.25$ and $\delta = 2.0 \pm 0.2$ for the size and duration distributions, respectively.

# 3 Supplementary Note 3: Waiting time distribution of pulses

The waiting time $t_W$ between consecutive pulses was determined as the duration of the silent period between them, i.e. the difference between the time of the first breaking of the later pulse and the time of the last breaking of the previous one. The probability distribution $p(t_W)$ of $t_W$ is presented in Fig. 3 for different system sizes $R$ and driving rates $s$. It can be observed that at a fixed driving rate $s = 0.04$ for larger systems the waiting times get shorter indicated by the decreasing cutoff value, and the distribution becomes steeper. Increasing the shrinking rate $s$ at a fixed system size $R = 90$ has a similar effect, i.e. at higher $s$ waiting times get shorter. The result implies that as pulses get larger for larger systems and higher shrinking rates the time gap between them gradually disappears so that in the limit of large system size a single pulse can be expected where all the cracking events merge into a giant pulse.

Our data analysis revealed that the waiting time distribution of pulses

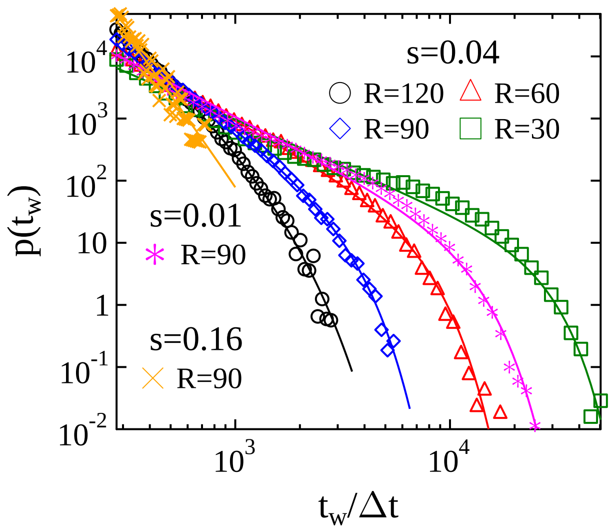


Supplementary Figure 3: Probability distribution of the waiting time $p(t_W)$ of pulses for different system sizes $R$ and shrinking rates $s$. The waiting time $t_W$ is made dimensionless dividing it by the time increment $\Delta t$ used in DEM simulations. The continuous lines represent fits with Eq. (S2).

can be well fitted by the functional form

$$p(t_W) \sim t_W^{-z} e^{-t_W/t_W^*}, \tag{S2}$$

where $t_W^*$ denotes the characteristic value of the waiting time which controls the cutoff of $p(t_W)$ (see Fig. 3). In agreement with the above arguments, the value of $t_W^*$ decreases while the exponent $z$ increases from 1.5 to 4.5 with increasing $sR^2$ indicating the disappearance of large waiting times.
